# Non-Invasive Placental Perfusion Imaging in Pregnancies Complicated by Fetal Heart Disease Using Velocity-Selective Arterial Spin Labeled MRI

**DOI:** 10.1038/s41598-017-16461-8

**Published:** 2017-11-23

**Authors:** Zungho Zun, Greg Zaharchuk, Nickie N. Andescavage, Mary T. Donofrio, Catherine Limperopoulos

**Affiliations:** 1 Division of Diagnostic Imaging and Radiology, Children’s National Medical Center, Washington, DC, United States; 2 Division of Fetal and Transitional Medicine, Children’s National Medical Center, Washington, DC, United States; 3 Department of Pediatrics, School of Medicine and Health Sciences, George Washington University, Washington, DC, United States; 4 Department of Radiology, School of Medicine and Health Sciences, George Washington University, Washington, DC, United States; 5 Department of Radiology, Stanford University, Stanford, California, USA; 6 Division of Neonatology, Children’s National Medical Center, Washington, DC, United States; 7 Division of Cardiology, Children’s National Medical Center, Washington, DC, United States

## Abstract

The placenta is a vital organ for fetal growth and development during pregnancy. Congenital heart disease (CHD) is a leading cause of morbidity and mortality in newborns. Despite the parallel development of the placenta and fetal heart early in pregnancy, very few studies suggested an association between placental dysfunction and fetal CHD. In this study, we report placental perfusion of healthy pregnancies and pregnancies complicated by fetal CHD measured using advanced fetal MRI techniques. We studied forty-eight pregnant women (31 healthy volunteers and 17 with fetal CHD) that underwent fetal MRI during their second or third trimester of pregnancy. Placental perfusion imaging was performed using velocity-selective arterial spin labeling (VSASL) and 3D image acquisition with whole-placenta coverage. In pregnancies with fetal CHD, global placental perfusion significantly decreased and regional variation of placental perfusion significantly increased with advancing gestational age; however, no such correlation was found in healthy pregnancies. Also, global placental perfusion was significantly higher in fetal CHD versus controls, in the lateral side-lying patient position versus supine, and in the posterior placental position versus anterior placental position. This study reports for the first time non-invasive whole-placenta perfusion imaging in utero. These data suggest that placental VSASL may serve as a potential biomarker of placental dysfunction in fetuses diagnosed with CHD.

## Introduction

The placenta is a vital organ that provides oxygen and nutrients, discharges wastes, and serves as an immune barrier for fetal well-being during pregnancy. Early in pregnancy, remodeling of the spiral arteries develops such that blood flow in the maternal-placental circulation increases in order to support rapid fetal growth and development^1^. When this remodeling fails, placental insufficiency occurs where oxygen and nutrients are not adequately supplied to the fetus through the feto-placental circulation^1–4^. While placental insufficiency is known to often result in fetal growth restriction (FGR)^1,2^ or preeclampsia^3,4^, the relationship between placental function and congenital heart disease (CHD) in fetuses is largely unknown. Despite the parallel development of the placenta and fetal heart early in pregnancy, very few studies have highlighted an association between the placenta and fetal CHD^5–9^. Notably, impaired placental growth in CHD has been shown to be associated with gestational age (GA) and birth weight at delivery, suggesting that abnormalities in placental development may contribute to increased morbidity in this high-risk population^8^. The vascular density, vascular area, and the number of terminal villi of the placenta have also been reported to be small in CHD based on placental pathology after delivery^9^. The vascular function of the placenta during pregnancy, however, is poorly understood namely due to the absence of non-invasive tools for measuring and monitoring placental function in utero.

Arterial spin labeling (ASL) is a powerful MRI technique for the direct, quantitative measures of regional tissue perfusion and has been extensively applied to the brain^10^. Unlike conventional perfusion MRI techniques, ASL does not require contrast agents because it utilizes the water molecules in arterial blood as an endogenous contrast agent. ASL is a particularly attractive method for early and safe monitoring during pregnancy given that ASL is completely non-invasive and does not require contrast agents or exposure to ionizing radiation. In addition, ASL provides reliable quantitative measurement, which enables comparison of absolute perfusion between different patients. To date, only two groups have applied ASL to the placenta and demonstrated limited ASL image quality and imaging coverage; this may be largely due to technical limitations of the labeling paradigm used in their studies^11–14^. No study has examined placental perfusion in fetuses diagnosed with CHD using ASL.

In this study, we used a novel placental perfusion imaging technique described by our group using velocity-selective ASL (VSASL) and 3D image acquisition to achieve higher sensitivity to placental perfusion and increased signal-to-noise ratio (SNR)^15^. VSASL allows for the labeling of all flowing arterial spins with a specific range of velocity, regardless of location^16,17^. Using VSASL, we measured total placenta perfusion that includes both maternal and fetal contributions. We quantified both global perfusion and regional perfusion variation in the placenta of pregnancies complicated by fetal CHD and healthy controls in order to determine whether placental perfusion alterations are evident in utero in the setting of fetal CHD.

## Results

### Study population

Among 50 pregnant women that we recruited, one pregnant woman complicated by fetal CHD and one healthy pregnant woman were excluded due to severe motion artifacts. Table 1 summarizes the baseline characteristics of our remaining study cohort (31 fetal CHD, 17 controls). The primary diagnosis for fetal CHD was used. Three out of 17 (18%) pregnant women with fetal CHD and 4 out of 31 (13%) healthy pregnant women received a second, follow-up fetal MRI scan using the same scan protocol. There was no significant difference in GA, maternal age, and fetal sex between CHD and healthy control fetuses. There were no maternal conditions in our study population except for one pregnant woman with fetal CHD who had gestational diabetes.

**Table 1:** Characteristics of our study cohort (n = 48).

Characteristics	CHD (n = 17)	Controls (n = 31)	p-value
Gestational age at MRI (weeks)	32 ± 5 (22–38)	30 ± 5 (21–39)	0.36
Maternal age (years)	33 ± 5 (23–43)	34 ± 4 (26–41)	0.70
Male fetuses	10 (59%)	14 (45%)	0.37
Subjects with two MRI scans	3 (18%)	4 (13%)	
Primary CHD diagnosis			
Hypoplastic left heart syndrome	7 (41%)		
Tetralogy of Fallot	3 (18%)		
Ventricular septal defect	2 (12%)		
Truncus Arteriosus	2 (12%)		
Double-outlet right ventricle	1 (6%)		
D-Transposition of the great arteries	1 (6%)		
Total anomalous pulmonary venous return	1 (6%)		

The p-values were estimated based on a Wilcoxon rank-sum test for GA and maternal age, and a chi-squared test for fetal sex.

### Global placental perfusion measures

**Figure 1:**
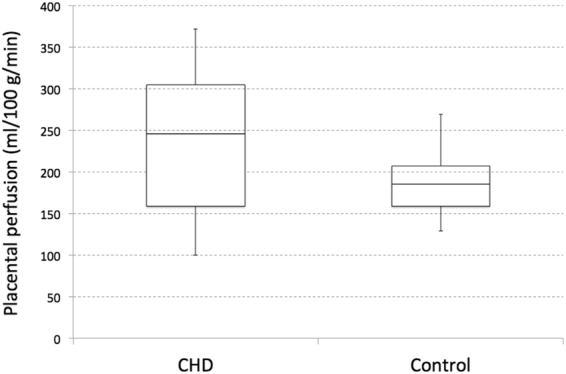
Box and whisker plot of global placental perfusion of pregnancies with fetal CHD and healthy pregnancies. When controlling for GA, there was a significant difference in global perfusion between CHD and controls (p < 0.01).

**Figure 2:**
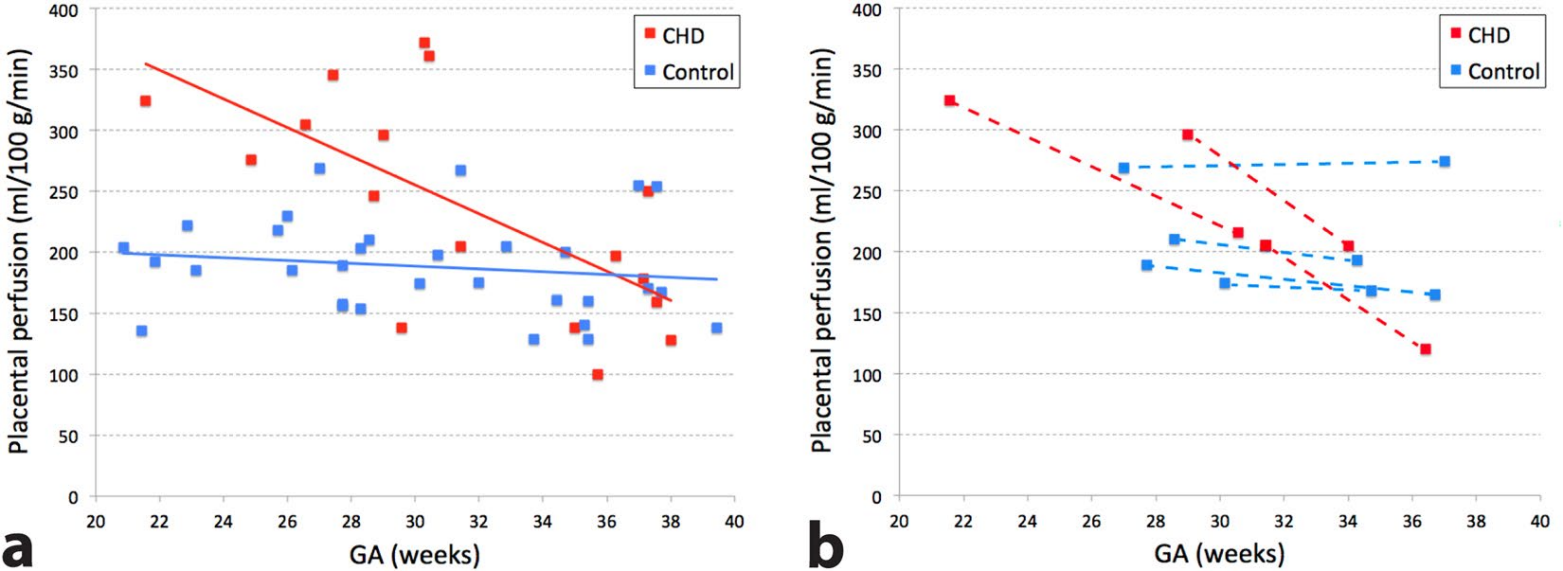
(**a**) Scatter plot of global placental perfusion against GA, including only the single or the first scan data. Only pregnancies with fetal CHD demonstrated a significant correlation between global placental perfusion and GA (r = −0.67, p < 0.01). (**b**) Two-time-point global perfusion data from seven subjects who received MRI scans twice on different days (first and second fetal MRI), with a dotted line connecting the two data points within each subject. The slope of the connecting line is akin to that of linear regression line of each subject group illustrated in (**a**).

Global placental perfusion, which we define as the mean placental perfusion of all voxels within the placenta of each subject, was found to be 236 ± 88 ml/100 g/min in pregnancies with fetal CHD and 188 ± 40 ml/100 g/min in healthy pregnancies (p = 0.10). As shown in Fig. 1, there was a substantially larger variation of perfusion in pregnancies with fetal CHD. After controlling for GA, global placental perfusion was significantly higher in CHD versus control placentas (p < 0.01). Interestingly, plotting global placental perfusion and GA revealed different global perfusion characteristics in pregnancies with CHD versus controls. Figure 2a shows a scatter plot of global placental perfusion and GA (excluding the data from the second fetal MRI). While healthy controls showed no significant correlation between global placental perfusion and GA (r = −0.16, p = 0.40), pregnant women with fetal CHD demonstrated a significant negative correlation between global perfusion and GA (r = −0.67, p < 0.01). Furthermore, data from 7 subjects (3 CHD and 4 controls) who underwent fetal MRI scans twice are displayed in Fig. 2b. The interval between the first and second fetal MRI scans was 5, 5, and 9 weeks for the three fetuses with CHD, and was 4, 6, 9, and 10 weeks for the four controls. The slope between the two data points within each subject was −18.2, −17.0, and −12.0 for CHD and −1.3, −3.0, −2.7, and 0.5 for controls (all in unit of ml/100 g/min/week). These were similar to the slope of the linear regression line in each group, which was −11.8 and −1.1 ml/100 g/min/week for CHD and controls respectively. The CHD diagnoses of these three subjects were as follows: two hypoplastic left heart syndrome (HLHS) and one tetralogy of Fallot (TOF).

### Relationship between placental perfusion and i) patient position during the fetal MRI and ii) position of the placenta

**Figure 3:**
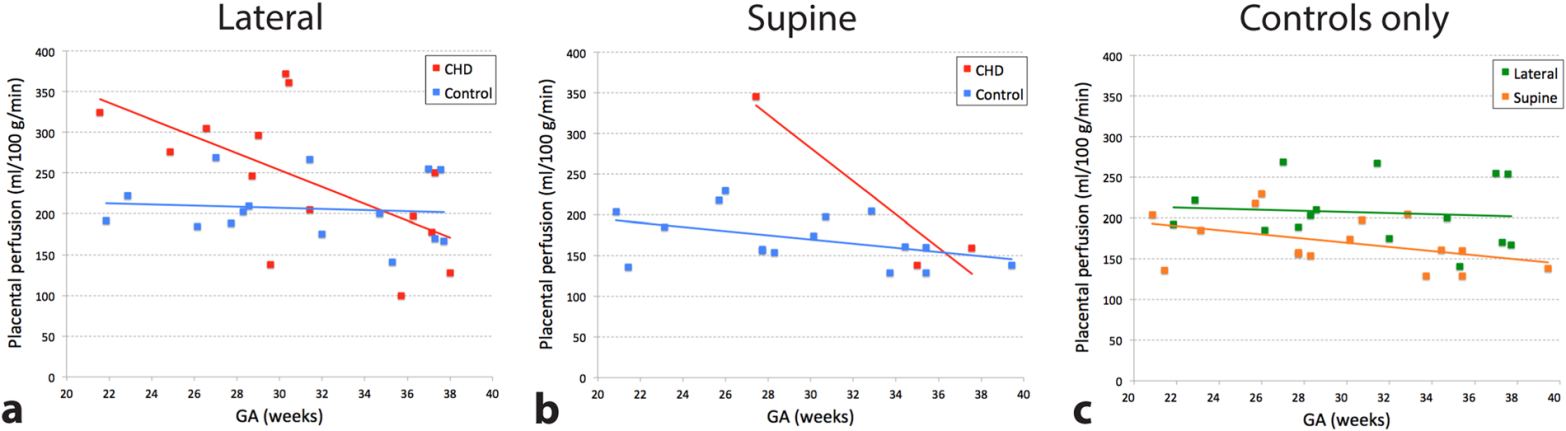
Global placental perfusion stratified on the basis of the patient position during the fetal MRI. (**a**) Lateral position: Although similar correlations are noted as those observed in the overall cohort, global perfusion in healthy pregnancies demonstrated even more consistent global perfusion across GA (r = −0.09, p = 0.74). (**b**) Supine position: Healthy controls showed a decreasing trend in global perfusion with advancing GA (r = −0.43, p = 0.09). (**c**) Healthy control data of lateral and supine patient positions are compared side-by-side, showing a significant difference in global placental perfusion (p < 0.01).

We compared global placental perfusion in relationship to patient position during the fetal MRI (see Fig. 3) given that the gravid uterus may reduce placental perfusion in the supine position^18^. In our data, 14 out of 17 pregnant women with fetal CHD (82%) and 15 out of 31 healthy pregnant women (48%) were scanned in the lateral side-lying position while the remaining pregnant women were scanned in the supine position. In pregnancies complicated by fetal CHD, global placental perfusion acquired in the lateral position showed a significant correlation with GA (r = −0.61, p = 0.02). This is similar to the result from overall fetal CHD data of lateral and supine positions because the difference in sample size was only three. Healthy controls, however, were divided almost equally into data acquired in the lateral (n = 15) and supine (n = 16) positions. While global placental perfusion of the healthy controls acquired in the lateral position was relatively consistent across GA (r = −0.09, p = 0.74), healthy controls studied in the supine position showed a negative trend in global placental perfusion with advancing GA (r = −0.43, p = 0.09). Mean global placental perfusion of healthy controls studied in the lateral and supine positions were 207 ± 39 ml/100 g/min and 171 ± 32 ml/100 g/min, respectively (p < 0.01 controlling for GA). Furthermore, we divided the subjects based on their position of the placenta in the uterus (anterior or posterior). For fetal CHD, 10 out of 14 women that were studied in the lateral position had an anterior placenta (71%), and 2 out of 3 women that were studied in the supine position had an anterior placenta (67%). The healthy pregnant women that were studied in the lateral and supine patient positions were both divided into anterior and posterior placental position almost equally (8 anterior vs 7 posterior for lateral, 8 anterior vs 8 posterior for supine). Because the sample size was small and unbalanced in fetal CHD, we focused on healthy controls for further analysis. Using multiple linear regression analysis (independent variables: GA, patient position, placental position), global placental perfusion of healthy controls was found to be significantly higher in the lateral patient position versus supine (p < 0.01) and in the posterior placentas versus the anterior placentas (p = 0.01). These results were more pronounced compared to when patient position (p = 0.01) or placental position (p = 0.04) alone was included in the analysis.

### Regional placental perfusion measures

**Figure 4:**
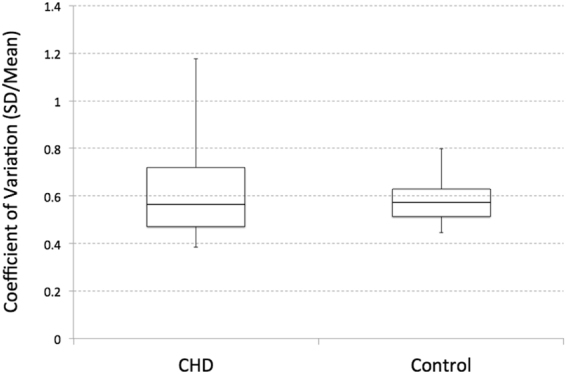
Box and whisker plot of coefficient of variation (SD/mean) of segmented placental perfusion of pregnancies with fetal CHD and healthy pregnancies. No significant difference between the two groups was found when controlling for GA (p = 0.50).

**Figure 5:**
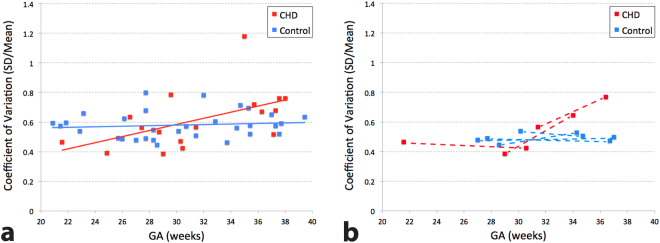
(**a**) Scatter plot of the coefficient of variation of segmented placental perfusion and GA, using the single or first scan data. Significant correlation between coefficient of variation and GA was found only in pregnancies complicated by fetal CHD (r = 0.53, p = 0.03). (**b**) Data from subjects who received two MRI scans, with a dotted line connecting the two data points within each subject. The slope of each subject is similar to that of the linear regression line from its own group shown in (**a**) except for one subject with fetal CHD.

The coefficient of variation of the segmented placental perfusion that represents spatial variation of regional perfusion, was found to be 0.62 ± 0.20 in pregnancies with fetal CHD and 0.58 ± 0.10 in healthy pregnancies (see Fig. 4). There was no statistically significant difference in the coefficient of variation between the two groups when controlling for GA (p = 0.50). Again, however, the scatter plot (Fig. 5a) showed a close association of the coefficient of variation and GA in pregnancies with fetal CHD only (fetal CHD: r = 0.53, p = 0.03 vs controls: r = 0.11, p = 0.55). With the exception of the outlier at GA of 35 weeks, fetal CHD showed an even higher correlation with GA. While global placental perfusion decreased with advancing GA in pregnancies with fetal CHD, regional perfusion variation increased with advancing GA in this group. The slope of the linear regression line was 0.02/week for CHD and 0.00/week for controls. Figure 5b shows data from seven subjects who underwent two fetal MRI scans. The slope between the two data points within each subject was 0.05, 0.04, and 0.00/week for the three pregnant women with fetal CHD and was −0.01, 0.01, 0.00, and 0.00/week for the four healthy controls respectively. Except for the slope of 0.00/week in fetal CHD, these slopes from both fetal CHD and controls showed a similarity to the regression lines of their own groups. Unlike global placental perfusion, there was no significant difference in regional variation between lateral and supine patient positions or between anterior and posterior placentas when controlling for GA, and thus patient position and placental position were not included in this analysis.

**Figure 6:**
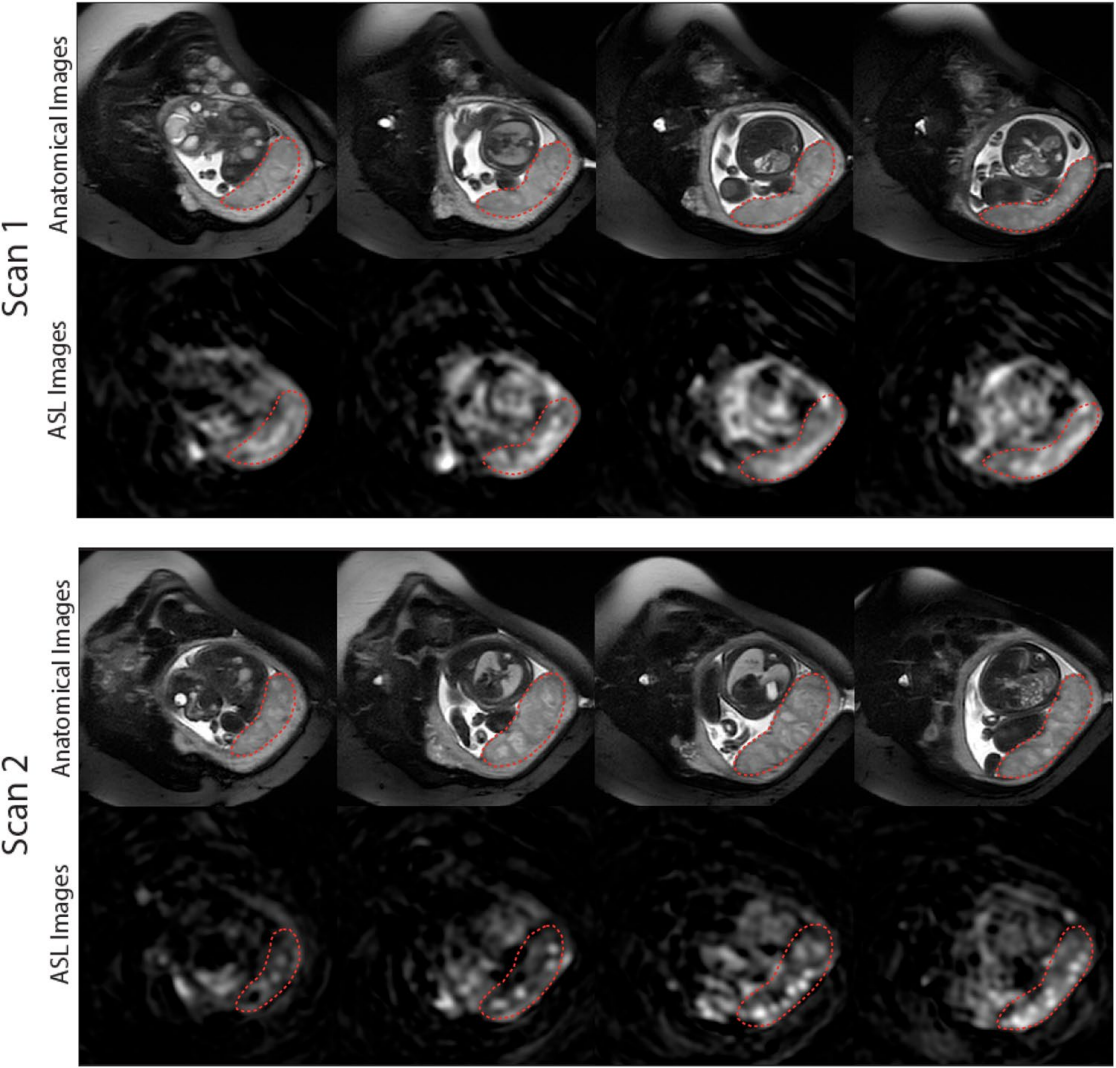
ASL and anatomical MR images of a pregnant woman with fetal CHD (diagnosis: HLHS) in the first (GA: 29 weeks) and follow-up (GA: 34 weeks) MRI scans. The placenta is delineated with the dotted line on each image. In the follow-up scan, ASL signal decreased in the regions outside lobules within the placenta, leading to increased regional variation of placental perfusion.

Figure 6 contains the ASL images of the first and follow-up scans from one representative pregnant patient complicated by fetal CHD (diagnosis: HLHS) in the selected slice locations. T2-weighted anatomical images on the corresponding slice locations are also displayed as a reference. The ASL images demonstrate that the ASL signal in the placenta was more homogeneous in the first fetal MRI scan compared to the follow-up scan, although the first scan already started to show mild lobulated patterns on ASL images. This lobulation on the ASL images became more conspicuous in the follow-up MRI scan; while the lobules contained most of the signal intensity, the regions outside the lobules suffered from substantial signal reduction. Similarly, it can be observed that lobulation was also present on the anatomical images, but not as dramatic as seen on the ASL images.

## Discussion

In this study, we successfully demonstrated non-invasive placental perfusion quantification in 17 pregnant women whose fetuses were diagnosed with CHD and 31 healthy pregnant women using 3D VSASL MRI. We analyzed global perfusion and regional perfusion variation as a function of GA, and reported that changes in placental perfusion were significantly related to advancing GA in CHD fetuses, but not in healthy controls. In pregnancies complicated by fetal CHD, global placental perfusion significantly decreased and regional variation of placental perfusion significantly increased with advancing GA. These findings may be beneficial for detection of early functional placental perturbations in fetal CHD. In addition, global placental perfusion was higher in pregnant women studied in the lateral patient position versus supine during the fetal MRI and in women with the posterior placentas versus the anterior placentas. To the best of our knowledge, this is the first study that demonstrates perfusion imaging of the whole placenta *in vivo*, the first study that utilizes VSASL in the placenta, and the first to provide evidence of second and third trimester placental perfusion differences between pregnancies complicated with fetal CHD and healthy pregnancies.

The labeling method of VSASL has several advantages for placental imaging compared to conventional ASL techniques such as pulsed ASL (PASL)^19,20^ or pseudocontinuous ASL (PCASL)^21^. First, VSASL is well suited for organs like the placenta that have large intravascular space (i.e. small extravascular). Conventional ASL schemes rely on the water being extracted from blood and being trapped in tissue with a long mean transit time due to large extravascular space in most of human tissues, similar to the microsphere technique. With these methods, significant ASL quantification errors can occur for the placenta where water exchange between blood and tissue is limited due to its relatively small extravascular space. In contrast, VSASL requires no water exchange because there is no need for a long post-labeling delay afforded by nominally zero arterial transit delay, and therefore VSASL can be successfully applied in such tissue types. This has been demonstrated previously in the lung, which is another human organ with short mean transit time^22^. Second, spatially non-selective labeling of VSASL is highly beneficial for placental ASL. The arterial blood path from the iliac artery to the uterine artery, which is the major route for the arterial blood supply to the utero-placental circulation, is extremely tortuous. Furthermore, there are other contributions to the placental circulation such as ovarian blood flow, which adds up to 15% of the total placental perfusion^23^. With PCASL, it may not be feasible to find a single labeling plane that captures all of the feeding arteries unless the labeling plane is placed in the descending aorta, which in turn would be far from the placenta, leading to significant SNR reduction. In addition, even delineating a suboptimal labeling plane in each subject would be very time-consuming or may require MR angiography prior to the ASL scan. PASL, on the other hand, is also not optimal for placental ASL because the feeding arteries needed for labeling are likely to be included in the target imaging slab, leading to compromised labeling. While these limitations of labeling efficiency may be resolved and are less problematic in single-slice imaging, single slice imaging in turn, limits its clinical use. For these reasons, whole-placenta ASL imaging may be achievable only with VSASL among current ASL techniques.

Mean global placental perfusion measured in normal pregnancies using VSASL in our study was found to be 188 ml/100 g/min (207 and 171 ml/100 g/min in the lateral and supine positions respectively) and it was consistent across GA, which corroborates previous studies. Placental ASL was first reported almost twenty years ago from a group that published three different studies using presumably the same imaging sequence based on PASL^11–13^. Gowland *et al*. demonstrated placental ASL with an average perfusion value of 176 ml/100 g/min in normal pregnancies (n = 16) and showed no correlation between placental perfusion and GA^11^. Duncan *et al*. reported placental perfusion of 209 ml/100 g/min in normal pregnant women (n = 45) with no correlation with GA^12^. Francis *et al*. showed a histogram of pixel fraction of placental perfusion that ranged between 0 and 1000 ml/100 g/min in healthy pregnancies (n = 6)^13^. Despite the small sample size, these are comparable to our measurements. Patient positioning was not taken into account in these studies. Recently, Derwig *et al*. reported placental ASL using a PASL technique more optimized for humans, but presented an analysis of ASL signal only, without absolute perfusion quantification^14^.

Our results are also comparable to reported values of placental blood flow acquired via Doppler sonography. Doppler sonography quantifies volumetric blood flow (in ml/min) by measuring vessel diameter and mean velocity of blood flow. Flo *et al*.^24^ and Rigano *et al*.^25^ reported measurements of volume blood flow in the uterine arteries of normal pregnant women throughout the second half of pregnancy and both groups demonstrated a significant increase of uterine blood flow during this period of pregnancy, showing good agreement between each other. Uterine blood flow in the unit of ml/100 g/min can be estimated by dividing the volumetric blood flow by placental weight that was reported in ref.^26^. Based on these published data, we estimated that uterine blood flow normalized by placental weight to decrease from 137 to 104 ml/100 g/min and from 105 to 83 ml/100 g/min during the second half of pregnancy based on refs^24,25^, respectively. Likewise, the contribution of umbilical artery flow to placental perfusion in normal pregnant women was estimated in the same manner and was found to be rather consistent over GA with the perfusion value of approximately 40 ml/100 g/min^27–29^. Given that perfusion measurement with VSASL includes both contributions of uterine and umbilical arteries, our results should be compared with the sum of these two perfusion values, which was slightly lower than that of our healthy controls. However, our measurement with VSASL may have also included contributions of ovarian arteries and other collateral vessels that may contribute to the placental circulation.

We further analyzed placental perfusion measures of pregnant women studied in the lateral and supine positions during the fetal MRI, and demonstrated a significant difference in placental perfusion between the two patient positions. It has been recommended that the supine position of pregnant women should be avoided for lengthy fetal MRI studies because maternal blood circulation is likely disturbed by compression of the inferior vena cava due to the mass effect of the gravid uterus^18^. Our data support that this effect may also influence placental perfusion. In our healthy controls, placental perfusion was not only significantly lower in the supine position compared to the lateral position, but while perfusion was stable in the lateral position, there was a progressive decrease in perfusion with advancing gestation in the supine position, likely due to increasing weight of the gravid uterus over time. These findings recommend the use of lateral decubitus position for the measurement of placental perfusion to avoid the mass effect of the gravid uterus. In addition, placental perfusion was found to be significantly higher in the posterior placentas versus anterior. Only one previous study has examined the relationship between anterior and posterior placentas, they showed no significant difference placental position and pulsatility index of the uterine arteries^30^. However, it is important to note that pulsatility index may not be correlated with placental perfusion. These intriguing preliminary findings in our study need further validation on a larger cohort which is currently underway.

Our findings demonstrated that both global placental perfusion and regional variation of placental perfusion in fetal CHD were significantly different compared to controls. Similar to the human brain, heart, and kidney, the placenta may have an autoregulatory mechanism to maintain optimal perfusion^9^. The early increased global placental perfusion in fetal CHD observed in our study may represent an attempt to compensate for the perfusion deficit in the fetal circulation, which may eventually fail. On the other hand, reduction of placental perfusion later in pregnancy among fetuses with CHD often revealed a lobulated pattern on the perfusion maps of our data. As previously observed on T2-weighted images^31,32^, these lobules may correspond to cotyledons of the placenta (i.e. fetal compartment) and the regions outside the lobules correspond to the septa, which represent parts of the decidua (i.e. maternal compartment) extended toward the intervillous space. Reduced perfusion in the maternal compartment of the placenta may suggest that the balance of placental perfusion is adjusted in response to an abnormal fetal circulation, and more blood flow is directed to the fetus rather than the placental tissue itself. On T2-weighted images, visible lobulations later in pregnancy may be explained by the T2 differences between blood and placental tissue^33^ given that blood volume, which is likely correlated with perfusion, may also decrease in the maternal compartment. Interestingly, several CHD cases in our study exhibited placental lobulation patterns on ASL images that were not evident on T2-weighed images, suggesting that functional lobulations in the placenta may be detected by ASL images prior to the anatomic changes detected on T2-weighted images. These global and/or regional perfusion changes across GA may have the potential for developing a future early biomarker of placental dysfunction in fetal CHD. Once placental dysfunction is detected before placental abnormalities become irreversible, there will be new, currently unavailable opportunities for potential treatment and preventive strategies. Ongoing studies are needed to address this important research question.

Placental ASL may play an important role in investigating potential causes and morbidities associated with CHD. Our findings suggest that fetal CHD is associated with placental dysfunction that is evident in the second and third trimester of pregnancy. This is corroborated by prior studies that demonstrated reduced oxygenation in the umbilical vein and the placenta or an increased incidence of placental insufficiency (such as FGR and preeclampsia), in the setting of fetal CHD^34–37^. Huhta and Linask suggested that placentation and fetal heart development may be affected by common environmental factors that predispose to malformation^5,6^. *In-vivo* monitoring of the placenta using ASL early in pregnancy may help elucidate the shared etiology of CHD and placental dysfunction.

Although there are a number of strengths in our study, the limitations deserve mention. First, our preliminary data come from a relatively small sample of healthy and high-risk pregnant populations, which included a heterogeneous cohort of CHD diagnostic groups. We did not find a significant difference in placental perfusion between single-ventricle and two-ventricle diseases or between cyanotic and non-cyanotic CHD (data not shown). Nonetheless, a larger sample size is warranted to confirm these initial findings. Second, placental perfusion measured using VSASL included contributions of both maternal and fetal circulation. Although this may not necessarily be a limitation, it would be of interest to determine which contribution (fetal vs maternal) represents the major source of altered placental perfusion in this high-risk population. Such investigations may be feasible with the development of new imaging schemes that measure each contribution separately. These studies are currently underway.

In conclusion we report for the first time, *in-vivo* whole-placenta perfusion imaging in pregnant women using VSASL-MRI. Our preliminary results show that global placental perfusion decreases and regional variation of placental perfusion increases with advancing GA in CHD fetuses only. We also report that global placental perfusion is significantly higher in the lateral patient position versus supine during the fetal MRI and in pregnant mothers with the posterior placentas versus anterior. Our preliminary findings suggest that placental perfusion is altered in pregnancies diagnosed with fetal CHD. The predictive value of these initial placental perfusion alterations on pregnancy and CHD outcomes are unknown and currently under study.

## Materials and Methods

### Study population

In the context of a prospective observational study, 18 pregnant women with a diagnosis of fetal CHD confirmed by echocardiography were recruited from the Fetal Cardiac Clinic at Children’s National Medical Center. Thirty-two control fetuses were studied from healthy pregnant volunteers with a normal prenatal history, and normal screening laboratory/ultrasound studies. Exclusion criteria for both healthy fetuses and fetuses diagnosed with CHD included: multiple gestation pregnancies, congenital infection, documented chromosomal abnormalities, and/or CNS and extra-CNS anomalies. Pregnant women who were unable to enter the MRI scanner for health (physical or psychological) reasons, or who did not meet standard criteria for MRI safety, such as the presence of non-removable ferromagnetic material were also excluded from the study. Enrolled pregnant women completed a fetal MRI study during the second or third trimester of pregnancy. A small subset of the study participants received two fetal MRI studies. This study was approved by the institutional review board of Children’s National Medical Center and written informed consent was obtained from each subject. All experiments were performed in accordance with the institutional guidelines and regulations.

### Fetal MRI

All MR imaging was performed on a GE MR450 1.5 T scanner using an 8-channel cardiac array coil. Pregnant subjects were scanned either in the supine or lateral decubitus position depending on the patient size and their preference. In each study, 2D T2-weighted single-shot fast spin echo imaging was performed for anatomical imaging and 3D VSASL was performed for the measurement of placental perfusion. This approach of placental perfusion imaging has been previously described and demonstrated to be superior in comparison with conventional ASL in our preliminary study^15^. T2-weighted imaging utilized the scan parameters of TR/TE = 1200/160 ms, matrix size = 256 × 192, FOV = 36–42 cm, and slice thickness = 4 mm with 40–60 slices. In VSASL, velocity-selective labeling was achieved using dual hyperbolic secant (sech) inversion with a cutoff velocity = 2 cm/s and TI = 1600 ms. Image acquisition in VSASL was performed using fast-spin-echo 3D stack of spirals with 8 interleaves, TR/TE = 3000/10 ms, in-plane matrix size = 64 × 64 over FOV = 36–40 cm, and slice thickness = 4 mm with 36–44 slices. Background suppression was achieved using one saturation and four sech inversion pulses. VSASL scan was composed of five pairs of control/labeled imaging followed by proton-density imaging, leading to total scan time of 4:30 min.

### Global placental perfusion measures

We used Buxton’s general kinetic model to quantify placental perfusion from ASL difference images and proton-density images^38^. Based on this model, placental perfusion (in ml/100 g/min) was calculated using the equation of *[6000·λ·(SI*_*control*_ − *SI*_*labeled*_ *)]/[TI·SI*_*PD*_ *·exp(*−*TI/T*_*1,blood*_ *)]* where *λ* is the tissue-blood partition coefficient (0.9 ml/g), *SI*_*control*_ and *SI*_*labeled*_ are the signal intensities in the control and labeled images, *TI* is the time between labeling and imaging, *SI*_*PD*_ is the signal intensity of the proton density image, and *T*_*1,blood*_ is T_1_ of blood (1.350 sec at 1.5 T)^10^. For calculating global placental perfusion, we manually delineated the placenta on each slice of the proton density images and averaged perfusion of all voxels within the whole placenta of each subject. The structural images were also used as a reference when the contrast of the placenta on proton density images was not clear.

### Regional placental perfusion measures

**Figure 7:**
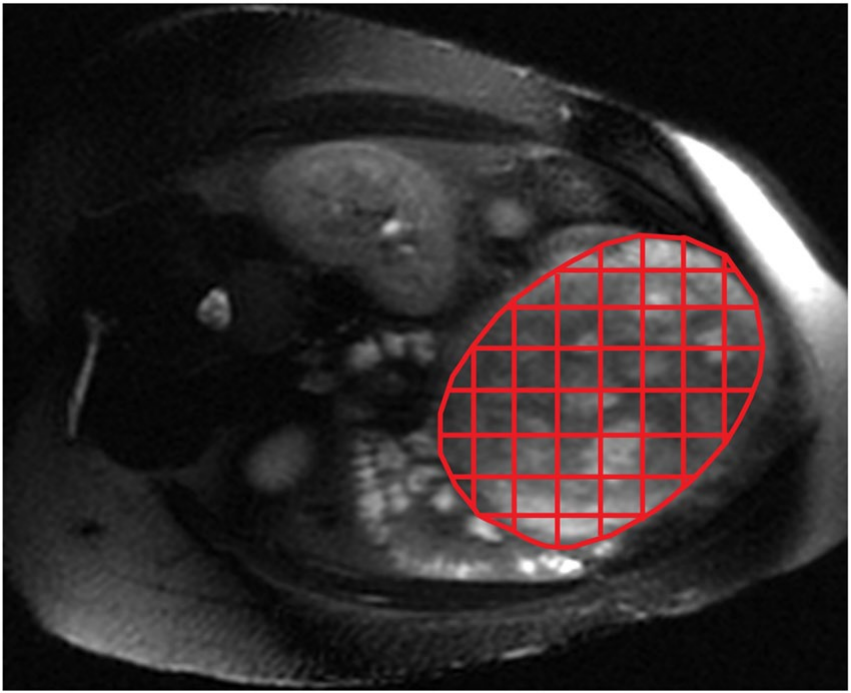
Placental segmentation with grid lines overlaid on an anatomical image for regional perfusion analysis. Partial segments on the boundaries were excluded from the analysis.

We postulated that impaired global perfusion in the compromised placenta would not appear as a global scaling effect, but rather be driven by regional perfusion reductions in the placenta. This can be predicted based on the typical lobulated pattern of the vascular development in the placenta, which also becomes evident on T2-weighted MR images with advanced GA or pathology^31,32^. To evaluate regional variation of placental perfusion, we extended our global perfusion analysis as follows. After the whole placenta was segmented for each subject, the placenta on each image slice was divided into multiple equal-sized segments, with the segments on the boundary of the placenta discarded (see Fig. 7). We used the segment size of 3 × 3 voxels from 128 × 128 interpolated ASL images, leading to segment area of 8.4–9.4 mm^2^ depending on FOV used for each subject. We measured mean perfusion within each segment and calculated a coefficient of variation (i.e. standard deviation divided by mean) of segmented perfusion measurements for each subject.

### Statistical analysis

Descriptive statistics were used to characterize our cohort. We used a Wilcoxon rank-sum test to compare GA and maternal age and a chi-squared test to compare fetal sex, between pregnancies with fetal CHD and healthy pregnancies. To determine the difference in global/regional placental perfusion between fetal CHD and controls, we used multiple linear regression analysis. We also calculated Pearson’s correlation coefficients and corresponding p values to examine the correlation between placental perfusion and GA. We used a significance level of 0.05 for all tests.
